# The impact of poverty on the development of brain networks

**DOI:** 10.3389/fnhum.2012.00238

**Published:** 2012-08-17

**Authors:** Sebastián J. Lipina, Michael I. Posner

**Affiliations:** ^1^ Unidad de Neurobiología Aplicada, Centro de Educación Médica e Investigaciones Clínicas Norberto Quirno (CEMIC), Consejo Nacional de Investigaciones Científicas y Técnicas CONICETBuenos Aires, Argentina; ^2^ Centro de Investigaciones Psicopedagógicas Aplicadas CIPA, Universidad Nacional de San Martin (UNSAM)San Martín, Argentina; ^3^ Department of Psychology, University of OregonEugene, OR, USA

**Keywords:** childhood poverty, brain networks, plasticity, attention, literacy, numeracy

## Abstract

Although the study of brain development in non-human animals is an old one, recent imaging methods have allowed non-invasive studies of the gray and white matter of the human brain over the lifespan. Classic animal studies show clearly that impoverished environments reduce cortical gray matter in relation to complex environments and cognitive and imaging studies in humans suggest which networks may be most influenced by poverty. Studies have been clear in showing the plasticity of many brain systems, but whether sensitivity to learning differs over the lifespan and for which networks is still unclear. A major task for current research is a successful integration of these methods to understand how development and learning shape the neural networks underlying achievements in literacy, numeracy, and attention. This paper seeks to foster further integration by reviewing the current state of knowledge relating brain changes to behavior and indicating possible future directions.

## INTRODUCTION

The study of the influence of material and social deprivation on the central nervous system (CNS) has been an issue of interest in the agenda of neuroscience since the first half of the twentieth century. Early neuroscience studies analyzed how the exposure to complex, standard, or deprived environments modifies the brain of experimental animals (Hebb, 1949; Mohammed et al., 2002; Grossman et al., 2003; Markham and Greenough, 2004; Sale et al., 2008).

The development of neuroimaging has provided non-invasive methods for examining changes in gray and white matter of the human brain (Posner and Raichle, 1994; Raichle, 2009) and may now serve to better integrate animal work with the century long efforts to specify lifespan changes in critical areas of development. Applying neuroscience methods to issues of child poverty has emerged from these developmental efforts (Hackman and Farah, 2009; Lipina and Colombo, 2009; Hackman et al., 2010). Since the mid 1990s different researchers have used behavioral and imaging to compare the cognitive and academic performance of children with disparate socioeconomic status (SES; e.g., Korenman et al., 1995; Guo, 1998; Lipina et al., 2004, 2005; Mezzacappa, 2004; Noble et al., 2005; Farah et al., 2006). Advances in neuroimaging have made it possible to incorporate neural network analysis in studies of the influence of poverty (e.g., Noble et al., 2007; D’Angiulli et al., 2008; Raizada et al., 2008; Stevens et al., 2008, 2009; Kishiyama et al., 2009).

A major achievement in the use of fMRI to study human development has arisen through the study of brain connectivity at rest (Fair et al., 2009, 2011; Gao et al., 2009). The results to date have shown evidence of sparse connectivity between brain structures during infancy and a strong increase in long range connectivity at 2 years (Gao et al., 2009) and later (Fair et al., 2007, 2009). In studies of neonates, the parietal areas, prominent in the orienting of attention network, show strong connectivity to lateral and medial frontal areas. The mid frontal cortical area and the anterior cingulate cortex (ACC), which have been implicated in self-regulation (Posner and Rothbart, 2007b) and down-regulation of the amygdala during the processing of threatening social cues (McEwen and Gianaros, 2010), show a marked increased connections to frontal areas and to lateral parietal areas during childhood. In older children (Fair et al., 2009), these tendencies continue and the ACC becomes increasingly differentiated from the orienting network as one approaches adulthood. One view of control systems was suggested recently by Fair et al. (2011). They argued, “data suggested that there might be at least two control networks functioning in parallel. Based on the differences in their functional connectivity and activation profiles we suggested that each network likely exerts distinct types of control on differing temporal scales. The fronto-parietal network was proposed to be important for rapidly adaptive control and to work on a shorter timescale. The cingulo-opercular network was thought to be important for more stable set-maintenance, and to operate on a longer timescale. Since this initial work there have now been several reports supporting this framework.”

In the present paper, we specifically focus on the issue of neuroscience approaches to childhood poverty using the recent data on brain networks including the resting state data. The resting state studies provide a unique perspective on the developing human brain, but their weakness is the limited evidence on how these changes influence infant and child behavior. In the section of this paper on attention and self-regulation, we try to establish such links, trying to highlight what should be taken in consideration regarding plasticity sensitivity to learning in the first stages of development. The aim of our effort is to review current progress and identify target areas, which could help form a research agenda for the coming years. To carry this out we first examine the general plasticity of the nervous system from the cognitive neuroscience viewpoint. Then we consider in turn the neural networks underlying the regulation of stress, attention and self-regulation, literacy, and numeracy. These topics were chosen because of their importance in normal development and according to one recent review on the mechanisms of executive function and language as the most influenced by SES (Hackman and Farah, 2009; Lipina and Colombo, 2009; Hackman et al., 2010; Raizada and Kishiyama, 2010). We consider the integration of animal and human studies, imaging of both gray and white matter, and the role of genes and specific experience in developing brain networks. We concentrate on linking neuroscience methods with behavioral and psychological approaches to development. In the next to last section we review a number of other current approaches to these same issues. Since there is much we still do not know, in the final section we propose priorities for a research agenda to allow for further progress.

## BRAIN PLASTICITY

In recent years, neuroscience has begun to take seriously that experience shapes the brain in important ways. Plasticity has been used as a blanket term to cover these changes. One area of plasticity related to the possible consequences of poverty is that experimental studies on rodents and non-human primates exposed to motor, sensory, and social stimulation, show structural and functional changes in neuronal and non-neuronal components, in comparison with those subjects exposed to deprived environments (see a comprehensive approach to brain plasticity in Rosenzweig, 2003; Markham and Greenough, 2004; Sale et al., 2008; Lipina and Colombo, 2009). These changes include synaptic number and morphology, dendritic arborization, cell morphology, the number of astrocytes and glial-synaptic contacts, myelination, glial cell morphology; brain vasculature; brain cortex weight and thickness, rate of hippocampal neurogenesis, availability and metabolism of both neurotrophic factors and neurotransmitters in different brain areas, and neurotrophic and neurotransmitter gene expression as well.

Complex environments induce neuroanatomical and biochemical changes in several brain areas of young and adult rodents, including frontal, parietal, and entorhinal cortices, hippocampus, and cerebellum (Rosenzweig and Bennet, 1996; Mohammed et al., 2002; Grossman et al., 2003). A considerable body of literature links the hippocampus to various plasticity factors, and to learning and memory mechanisms, such as gene expression and protein levels of neurotrophins, glucocorticoid receptors, the Alzheimer amyloid precursor protein, immediate early genes, serotonin receptors, α-amino-3-hydroxy-5-methyl-4-isoxazolepropionic acid (AMPA) receptor binding, and neurogenesis (Mohammed et al., 2002). Middle-aged rats that had at least 1 year in complex environmental conditions showed higher levels of nerve growth factor (NGF) in the entorhinal cortex, compared to isolated animals (Mohammed et al., 2002). Furthermore, several studies suggest an involvement of these neurotrophins in the synaptic plasticity of the hippocampus and other brain areas (McAllister et al., 1995). Several neurotrophic factors, such as the NGF, the brain-derived neurotrophic factor (BDNF), and the neurotrophin-3 (NT-3), are abundantly expressed in pyramidal cells and dentate granule cells, a fact suggesting that neurotrophins are involved in mediating changes in the dendritic morphology following exposure to complex environments. In this regard, the role of the hippocampus in learning and memory has been the focus of increased research interest involving the generation of new neurons. Using the proliferation marker bromodeoxyuridine (BrdU), different studies showed that enrichment increases neurogenesis in the dentate gyrus of adult mice and rats. This effect has been associated with learning (Shors et al., 2001), while stress experience has been related to the reduction of neurogenesis (Gould et al., 1997).

The cerebellum has also been found to display plastic properties in response to environmental influences. Specifically, learning of a complex motor skill leads to an increase in the synaptic numbers of the cerebellar cortex, and to changes in the morphology of cerebellar Purkinje cells in rodents and monkeys (Kleim et al., 2003).

Behavioral changes following exposure to enriched environmental conditions are not limited to the improvement of cognitive performance. One robust observation has been based on the effects of isolation. Rodents after being reared in isolated environments interacted less with objects in a free exploration situation, while displaying at the same time an increased locomotor activity coupled to a reduced habituation. These findings would suggest that, depending on the complexity of environmental enrichment or deprivation, brain circuits would be affected with more, or less, specificity involving different cortical areas and subcortical structures. Although the experimental conditions of enrichment/deprivation cannot be identified directly with human poverty they may illuminate some aspects of child poverty (Lipina et al., 2011).

The findings of effects from enriched environments make useful interdisciplinary efforts and in particular to the translational animal and human research. For instance, in the context of analysis of the neural reactivity to stress the study of failures to habituate to repetition of the same stressor or to terminate adaptive autonomic and neuroendocrine response, are based upon the experimental animal–human translational efforts (McEwen and Gianaros, 2010).

Neural plasticity in humans may also lead to structural adaptation in cortical gray matter, in response to environmental demands (Bavelier and Neville, 2002). At the level of imaging studies, there is evidence that the brain may adapt dynamically to reflect environmental cognitive demands. Neuroimaging studies show evidence of structural changes in specific areas after training in difficult motor tasks. For example, studies of professional musicians (Gaser and Schlaug, 2003; Imfeld et al., 2009) show increased size of relevant motor areas, and selective increases in gray matter volume in posterior hippocampus. In addition, concomitant spatial memory performances have been shown in licensed London taxi drivers (Woollett and Maguire, 2011).

Current studies in the developmental neuroscience field continue to advance in the understanding of the plastic mechanisms through which experience and environmental influences interact with genes, more specifically with the DNA biochemical markers and histone proteins that regulate gene activity. Specifically, post-translational modifications of histones and DNA methylation are the most frequently analyzed mechanisms, involved in changes in gene activity with environmental factors (Zhang and Meaney, 2010; Roth and Sweatt, 2011). Learning and memory processes also evoke alteration of epigenetic markers in the adult CNS, as shown by animal models. For instance, Miller and Sweatt (2007) have used the contextual-fear conditioning paradigm to analyze epigenetic modulation of hippocampal genes. They found that during a period of fear memory formation, adult rats have a demethylation and transcriptional activation of the memory-enhancing gene reelin, and an increase in methylation and transcriptional silencing of the memory suppressor gene protein phosphatase 1.

These multiple changes in neural structure are correlated with functional changes in motor, cognitive, and emotional systems and behaviors (Mohammed et al., 2002; Grossman et al., 2003; Markham and Greenough, 2004). Thus, development and learning alter the local neural environment and that lead to the observed changes in brain and behavior (Galván, 2010).

For humans poverty is associated with a restricted environment and thus it would be expected that SES would be correlated with differences in brain and behavior. Researchers have reported that the modulation of performance by SES is not the same in all areas of behavior nor uniform at all ages (e.g., Lipina et al., 2004; Noble et al., 2005; Farah et al., 2006). Actually, SES is a multifaceted construct hard to capture by single or multifactor indexes, and each component (e.g., income, education, and occupation) represents resources that might benefit development in different ways (Duncan and Magnuson, 2012). Conceptually, this implies that poverty does not necessarily generate homogeneous and continuous changes in neurocognitive processing. These findings move away from the notion of low-SES performance as a deficit. Instead we need to develop measures for different brain networks; ages and SES/poverty conditions to contribute understand how poverty and SES shape brain networks development. For instance, Evans et al. (2005) have pointed to the role of chaotic unpredictable family environments – a specific aspect of SES, which is usually not analyzed – as a threat to emotion regulation. With the complexities of SES in mind developmental cognitive neuroscience may serve as fertile ground for new ideas about the role of SES in development.

In summary, the findings from behavioral studies indicate that SES disparities and poverty can adversely affect cognitive processes, such as language, executive function, attention, and memory. In addition, these findings suggest that specific brain regions are associated with these cognitive functions. However, these tests are still behavioral in nature and as such, they are subject to a number of limitations. For instance, researchers can make only indirect inferences about brain function from behavioral tests. In addition, many of these tests are multi-factorial and performance could be disrupted for reasons other than those resulting from a specific dysfunction. Moreover, correlations among these tests are low, which means that two tasks can engage the same system in different ways. So a deeper examination of the impact of SES and poverty on the relationship between cognitive processes and brain function is needed. To this end, neuroimaging techniques can be applied to analyze the neural level of analysis.

## BRAIN PLASTICITY AND STRESS

The stress system has been used as a model system for an examination of adverse early experience on a brain network (see Lupien et al., 2009 for a review). The basic brain network involved in the stress response is the hypothalamus–pituitary–adrenal (HPA) axis. It is vertically organized and involves the autonomic and the CNSs and hormonal as well as neural function.

Stress-related studies have been carried out in a variety of non-human animals as well as in humans (Lupien et al., 2009). These studies rely heavily on animal models to examine the effects of early environmental effects such as maternal care, caregiver maltreatment, mother–infant separation, and prenatal stress. The evidence suggests that early stress can produce lasting epigenetic modifications, stable changes in the CNS gene activity, as well as on behavior. For example, adult offspring raised by mothers providing high level of pup licking and grooming showed molecular changes in hippocampal glucocorticoid receptors, transcription of neural growth factor, corticotrophin releasing factor expressions, and glucocorticoid feedback sensitivity (Zhang and Meaney, 2010; Roth and Sweatt, 2011). Human studies by Roth et al. (2009) suggest that caregiver maltreatment can result in modified methylation of the BDNF in the prefrontal cortex, and a DNA hypermethylation, which paralleled a lasting deficit in expression of the gene. These effects could be treated in part by a DNA methylation inhibitor administered to adults. Similar findings in animal models resulted from maternal separation (Zhang and Meaney, 2010).

The epigenetic analysis of the early experiences on brain development in humans is in its first stages as many of the issues in the study of childhood poverty and brain development. For instance, recently McGowan et al. (2009) examined the gene expression and DNA methylation of the human glucocorticoid receptor (Nr3c1) gene in hippocampal samples from suicide victims with a history of childhood maltreatment. They found decreases levels of mRNA hippocampal glucocorticoid receptor gene, correlated with increases of cytosine methylation of the Nr3c1 promoter, which suggests that human caregiver experiences may program genes through epigenetic modifications. Bueller et al. (2006) have found evidences that carriers of the methionine allele of the Val66Met BDNF polymorphism express lower gray matter volume in the hippocampus and prefrontal cortex compared with carriers of the valine–valine allele, suggesting a pathway of modulation of BDNF secretion and intracellular functioning. In another study, Oberlander et al. (2008) found that infants of mothers with high levels of depression and anxiety during the third trimester of pregnancy had increased methylation of the Nr3c1 gene promoter in cord blood cells.

These studies support the hypothesis that the epigenome of prenatal developing infants is sensitive to the experiences of their mothers. Of course, many methodological issues should be explored in future studies, such as whether peripheral measures of DNA methylation accurately reflect CNS methylation (Roth and Sweatt, 2011).

In summary, we stress two important points from the work on stress. First it shows the importance of a distributed systems of brain areas in the control of stress, involving prefrontal cortex, hippocampus, amygdala, and the HPA axis (Lupien et al., 2009) which operate as a non-linear, interactive network in which multiple mediators regulate each other, and promote adaptive activities and coping with aversive situations and discrete stimuli, such as those usually present in low-SES and poverty contexts (e.g., crowding, hunger, threats to mental and physical health). These areas and the white matter connection between them are important contributors to the influence of stress on the person. Experimental animal models of the hippocampus have revealed a mechanism by which chronic stress leads to remodeling of hippocampus circuitry. These changes consist in shortening of dendrites, loss of spine synapses, and suppression of neurogenesis in the dentate gyrus. One of the effects of these processes is impairing hippocampal involvement in episodic, declarative, contextual, and spatial memory, what in turn leads to alter the ability to process information in new situations and to make decisions about how to cope with new challenges (McEwen and Gianaros, 2010).

Second, is the notion that epigenetic changes underlie the long-term impact of early experiences, and that epigenetic alterations are potentially reversible or modifiable through pharmacological and behavioral interventions. This means that the understanding of the role of genes and of the epigenome in behavioral modifications driven by early experiences could contribute to the field of childhood poverty and brain development. However, the presence of genetic variation in humans suggests that similar childhood experiences could produce different outcomes depending upon the exact version of the gene present in the individual. A child’s reaction to stress is an important factor in success in school and our understanding of the stress reaction may also guide us in analyzing other brain systems more directly involved in schooling as we try to do in the remaining sections of this paper.

## ATTENTION

Attention is a key factor in school readiness and success (Posner and Rothbart, 2007a). An understanding of the underlying brain networks involved in attention has been a major contribution from research using neuroimaging (Posner and Petersen, 1990; Posner and Rothbart, 2007b; Petersen and Posner, 2012). Attention networks are involved in obtaining and maintaining the alert state (alerting network), orienting to sensory stimuli (orienting network), and resolving conflict between responses (executive network). The executive network is a key to the role of ability of children and adults to regulate their thoughts and feelings. Adjacent areas of the ACC are involved in cognitive and emotional control (Bush et al., 2000). Connectivity of these control systems develop over the early life of infants and young children, and lead to the ability to regulate other brain networks, thus exercising executive control over behavior (Fair et al., 2007, 2009, 2011; Gao et al., 2009; Posner et al., 2012). This control depends critically upon factors in the social environment such as parenting. Better understanding of the mechanisms by which control develops and is exercised can provide guidance to parents and to society. Critical to this is an understanding of the mechanisms, which switch control from the caregiver during infancy to later self-regulation by the child.

One function that has been traced to the frontal midline (ACC) is monitoring and correction of errors. The ability to notice perceptual errors occurs as early as 7 months (Wynn, 1992; Berger et al., 2006) and activates the anterior cingulate in infants just as it does in adults. However, infants of this age are not able to use the information to control their behavior. We had children play a simple Simon game that asked them to execute a response command given by one puppet while inhibiting commands given by a second puppet (Jones et al., 2003). Children of 36–38 months showed no ability to inhibit their response and no slowing following an error, but at 39–41 months children showed both an ability to inhibit and slowing of reaction time following an error. These results suggest that between 38 and 39 months, performance changes based upon detecting an error response. These dramatic changes in error correction relate to the changing connectivity of the brain during early child development, as was indicated by studies with neonates and older children in which parietal areas related to orienting of attention show strong connectivity to lateral and medial frontal areas, and the ACC shows increased connections to frontal areas and to lateral parietal areas (Fair et al., 2009), suggesting tendencies of increasing differentiation in the networks involving parietal and frontal areas related to attentional processing.

However, changes in brain connectivity are not finished at age 3. The Attention Network Test (ANT) has been used to examine the efficiency of the three brain networks (Fan et al., 2002) in older children and adults. The task requires the person to press one key if the central arrow points to the left and another if it points to the right. Conflict is introduced by having flankers surrounding the target point in either point the same (congruent) or opposite (incongruent) direction as the target. Cues presented prior to the target provide information on where or when the target will occur. Reaction times for the separate conditions are subtracted, providing three measures that represent the efficiency of the individual in alerting, orienting, and executive networks.

We have examined the ANT in children from 6 to 10 years of age using a version specifically adapted to them. The results for children of this age are similar to those found for adults using the same child version of the task. The child reaction times are much longer, but they show similar independence between the three networks. Children have larger scores than adults for alerting up to age 10 and for conflict up to age 7, suggesting that young children have trouble in resolving conflict and even older children have trouble in maintaining the alert state when not warned of the target (Rueda et al., 2004).

## SES EFFECTS ON ATTENTION NETWORKS

In studies of preschoolers, first graders, and middle school children, low-SES children had reduced performance on many tasks compared to middle SES children (Noble et al., 2005; Farah et al., 2006). These findings suggest that the prefrontal/executive system is one of the primary neurocognitive systems associated with social inequalities in early experience. Similar results have been observed in studies using specific paradigms designed to measure aspects of both executive function and attention. For example, Lipina et al. (2005) examined performance of low and middle SES infants using a task of a delayed-response paradigm, which incorporates the evaluation of processes such as working memory and inhibitory control. Findings showed that low-SES infants made more errors associated with impairments of inhibitory control and spatial working memory, and errors associated with attention and search strategies.

The effects of socioeconomic disparities on attention have been examined in several studies. For instance, Mezzacappa (2004) used the ANT to investigate the effects of socioeconomic disparity on attentional processes in children of 6 years of age. This task can be used to assess alerting, orienting, and executive attention networks. Results showed that low-SES children had reduced measures of both speed and accuracy on measures of alerting and executive attention, indicating that SES modulated response conflict and inhibit distracting information.

Another report on the difficulty low-SES children have in attention comes from studies comparing the performance of high and low-SES children attempting to listen to a story presented to one ear while ignoring that on another ear. Performance is assessed by the amplitude of the P1 component of the auditory event-related potential (ERP) known to be influenced by attention. Low-SES children show little evidence of attention amplifying this EEG component while higher SES children of the same age show clear evidence of P1 amplification for the attended ear. After a period of training in classroom that provided practice in various attention networks the SES children also showed the influence of attention on P1 (Stevens et al., 2009; Neville et al., 2011).

## FORMS OF ATTENTION TRAINING

There has been considerable evidence that various types of attention training can be effective in children (Rueda et al., 2005, 2012; Diamond et al., 2007; Tang and Posner, 2009; Diamond and Lee, 2011; Neville et al., 2011; Klingberg, 2012; Segretin et al., 2012). Most of the evidence involves practice with attention related tasks either using computerized tasks or classroom curricula. Although it is likely that many of these methods target executive attention network, some may train primarily orienting to sensory stimuli. These methods have been shown to be effective in normal preschool children and in children with disorders such as ADHD or those with low-SES. It is not possible to say which methods are most effective. The computerized methods allow rather compete specification of the training, while the classroom methods provide more practical means of training. Most of the studies have not examined how long the training is effective, but one study suggested little loss after 2 months (Rueda et al., 2012). In general, many studies of early preschool education have shown that the advantages for specific skills taught in school disappear in a few years, but there is some evidence that general benefits of training of executive skills last for many years (Moffitt et al., 2011).

A second type of training involves changes in brain state that might influence some attentional networks as well as stress and immunoreactivity. For example, physical exercise has been shown to have general advantages for improving cognitive function in adults and elderly (Hillman et al., 2008) and meditation has been shown to have a specific influence on executive attention and stress in undergraduates (Tang et al., 2007). Meditation has been shown to be effective in children as young as 4 years of age (Tang et al., 2012), but the role of this method with low-SES children remains to be demonstrated.

## LANGUAGE AND LITERACY

Language and literacy are important in school and are functions found to be reduced in low-SES children. According to a recent analysis (Hackman et al., 2010) one of the systems most at risk for low-SES children involves language. In recent years, the way in which experience shapes language development starting in infancy has been analyzed in detail. Below we review some of these findings.

In the 1970s, behavioral studies using habituation to a repeated stimulus provided evidence that from birth infants are able to discriminate basic phonemes, the basic constituents of language, not only in their own language but also in other languages to which they have never been exposed (Eimas et al., 1971; Streeter, 1976). Studies using these behavioral methods together with electrical recording from the scalp have probed some of the early development of the phonemic structure underlying language. More recently, infants have been exposed to language while resting in fMRI scanners to examine the brain mechanisms activated by language (Dehaene-Lambertz et al., 2006).

The infant language system appears to involve the same left hemisphere language structures found in adults (Dehaene-Lambertz et al., 2006). In one study infants listened to sentences presented aurally in their language. Brain areas in the superior temporal lobe (Wernicke’s area) and in Broca’s area were active. When the same sentence was presented after a delay of 14 s Broca’s area activity increased, as though this area was involved in the memory trace.

It has long been supposed that the early acquisition of language might involve very different mechanisms than are active in adults (Vicari et al., 2000). Left hemisphere lesions in infancy do not produce a permanent loss of language function as they can in adults. Nonetheless, the new fMRI data suggests the left hemisphere speech areas are involved in receptive language even at 3 months of age and even though brain damage at this early age may allow the same functions to develop in the right hemisphere (Dehaene-Lambertz et al., 2006).

## PHONEMES

It has been possible to study changes in phonemic discrimination due to exposure to the native language at least by 10 months of age (Kuhl, 1994; Saffran, 2002). Infants appear to acquire a sharpened representation of the native phonemic distinctions (Kuhl et al., 2006). During this same period, they also lose the ability to distinguish representations not made in their own language (Werker et al., 1981). At least a part of the loss occurs when the non-native language requires a distinction that is within a single phonemic category in the native language. An example is the *ra–la* distinction important in English is lost because it is within a single category in Japanese (McClelland et al., 2002). It is as thought Japanese no longer hear this distinction and even when exposed to English they may not improve in distinguishing *ra* from *la*. In the McClelland et al.’s (2002) study an adaptive training regime starting with initially easy stimuli was contrasted with a fixed training regime using difficult stimuli, with some subjects receiving feedback on the correctness of their responses and others receiving no feedback in both conditions. After three and six sessions of training, subjects received tests assessing identification and discrimination of /r/–/l/ stimuli as well as generalization. In all cases except fixed training without feedback, subjects showed clear evidence of learning, and several indicators suggested that training affects speech perception, rather than simply auditory processes. Thus, training by several methods (McClelland et al., 2002; Iverson et al., 2005) seems to improve this form of phoneme discrimination even in adults, although it is not known how well this knowledge can be incorporated into normal daily life communication.

It might be useful to find a way that will preserve the distinctions originally made for the non-native language during infancy. One study showed that 12 sessions of exposure to a mandarin speaker during the first year of life help to preserve a mandarin phoneme in children whose native language was English (Kuhl et al., 2003). A similar amount of exposure to a computerized version of the speaker was not effective, suggesting the importance of social interaction in this early form of learning. More needs to be learned about how and whether media presentation can be effective in learning.

There are many reasons why it is useful to know more than one language. Much of the world population lives in places where speakers of two or more language live in close proximity. In addition, there is some reason to believe that proficiency in a second language provides improved performance in the ability to exercise executive control over thoughts (Bialystok and Martin, 2004), which might be one form of attention training.

There is also some reason to believe that the process of phonemic discrimination being developed in infancy is important for later efficient use of spoken and written language (Molfese, 2000; Guttorm et al., 2005), which is critical in childhood poverty studies since SES modulates the early language environments (Hoff, 2003). Electrical recording taken in infancy during the course of phonemic distinctions (Molfese, 2000; Guttorm et al., 2005) have been useful in predicting later difficulties in language and reading. There is a history of using ERPs to assess infant deafness early in life and being able to do so reliably have been very useful in the development of sign language and other interventions to hasten the infant’s ability at communication. Perhaps a similar role will prove to be possible for ERPs in the development of methods to insure a successful phonemic structure in the native language.

There have been efforts to develop appropriate intervention in later childhood for difficulties in the use of language and reading such as the widely used *FastForward* programs (Temple et al., 2003). Although there are disputes about exactly why and for what populations this method works it remains important to develop remedies for language difficulties based upon research.

## READING

Reading is a high-level skill and in alphabetic languages such as English, it has properties related to the phonemic structure of language. There have been many studies of adult reading and much more is known than can be reviewed here (see Posner and Rothbart, 2007a; Schlaggar and McCandliss, 2007 for reviews). Adult studies of reading have revealed a complex neural network involved in the translation of words into meaning. Two important nodes of this network are the visual word form area, of the left fusiform gyrus and an area of the left temporal–parietal junction for translating visual letters into sounds. The activation of the left temporal–parietal junction is modulated by SES, among other factors (Monzalvo et al., 2012).

Languages like English that are highly irregular in visual to sound mappings are heavily dependent upon brain areas that translate visual words to sound. Children who have difficulty in learning to read show little activation in these phonological areas (Shaywitz et al., 2007). Many of the studies of learning to read use dyslexic children who have shown specific difficulties in learning to read, some of what has been learned probably will apply to low-SES children as well, since the child’s ability in phonemic awareness, (e.g., rhyming of auditory words), is a good predictor of their being able to learn to read alphabetic languages such as English. Training studies designed to improve decoding have shown that children with low reading skill can improve and when this occurs they show enhanced activation in areas related to phonological translation (McCandliss et al., 2003a,b).

The visual word form area is involved in integrating or chunking visual letters into words (McCandliss et al., 2003b). Although there has been some dispute about its unique operations it appears to be a part of the visual system that becomes expert in dealing with letters as reading skill develops in later childhood (Price and Devlin, 2004; Ben-Shachar et al., 2011). It is thought that without the functioning of this area reading cannot become fluent. For example, patients with a lesion that interrupted the flow of information from the right hemisphere to the visual word form area used letter by letter reading when words were presented left of fixation (going to the right hemisphere), while they read words normally when the word was projected to the left hemisphere and thus reached the visual word form area (Cohen et al., 2004). Children from 7 to 18 who were deficient in both decoding and comprehension skills failed to activate this area, but were able to do so after extensive phonological training (Shaywitz et al., 2007).

The time course of development of the visual word form area in English is important for the development of fluent reading. Phonics training often leaves the child with improved decoding skill, but with a lack of fluency. Evidence that the visual word form develops rather late and first only for words with which the child is familiar (Posner and McCandliss, 1999), suggests the importance of continuous practice in reading to develop fluency (Shaywitz et al., 2007). More research is needed on the best methods for developing fluency particularly in non-alphabetic languages.

## LOW-SES CHILDREN LEARNING TO READ

A study by Noble et al. (2007), hypothesized that SES systematically influenced the relationship between phonological awareness skills and brain activity in areas involved in reading. Phonological awareness, as we discussed in the Section “Phonemes” has been a key predictor for success in learning to read. To test this hypothesis, researchers examined fMRI responses during a pseudoword reading task in first- to third-graders from diverse SES backgrounds. Results showed a significant phonological awareness–SES interaction in the left fusiform visual word form area, indicating that at similar low phonological awareness levels, children from higher SES were more likely to evidence increased responses in the left fusiform cortical gyrus, while children from lower SES did not.

In another recent study of healthy 5-year-old children performing an auditory rhyme-judgment task, Raizada et al. (2008) found a more direct relation: the higher the socioeconomic status, the greater the degree of hemispheric specialization in Broca’s area, as measured by the left-minus-right fMRI activation during rhyming tasks. This suggests that the maturation of Broca’s area in children may be governed by the complexity of the linguistic environments in which they grow up.

## SUMMARY

Language development begins very early in infancy. Exposure to language shapes the phonemic structure in a way, which could influence later acquisition of literacy. Imaging studies have traced the brain structure and connectivity involved in learning language and acquiring literacy. Interventions for improving brain areas in children with low learning skill have been developed and proven useful both for decoding of letters into their auditory form and for chunking letters into a visual unit. It seems likely that children in poverty face difficulty in all of these operations. Their exposure to reduced input of language during infancy may cause problems with perceiving phonemes, which in turn predicts performance in acquiring reading. Future studies may lead to further development of interventions designed to improve these skills in all children.

## NUMERACY

The human infant like other animals seems to have an inborn skill to recognize quantity. At least by a few months of age the infant seems to be able to discriminate changes when presented with a small number of events presented. Wynn (1992) showed that infants of 7–9 months looked longer when simple addition problems (presented as puppets) were in error than when they were correct. Berger et al. (2006) compared this ability in 7- to 9-month-old children and adults, using high-density electrical recording from the scalp. They found the same electrodes over frontal midline areas discriminated between errors and correct in both infants and adults. The adult brain made the discrimination by about 250 ms and the infant brain was only delayed by about 50 ms. The authors showed that error detection was signaled by an increase in theta rhythm in both groups. The electrodes in question had been related to activity in the dorsal ACC in previous studies (Dehaene et al., 1994).

The overall network of brain activity in processing number has been studied in children and adults by high-density electrical recording in a task which required the person to indicate by pressing keys whether a digit was above or below 5 (Dehaene, 1996; Temple and Posner, 1998). Children as young as 5 years of age showed similar brain mechanisms underlie the decision as found in adults suggesting that the number line can be used by this age. There has been some dispute concerning the developmental course of the number line as some studies have suggested frontal structures (Ansari et al., 2005), rather than parietal structures mediate this decision (Cantlon et al., 2006).

Apparently, there are linguistic and cultural differences in the use of Arabic digits in the performance of calculations that could have important consequences for the acquisition of language by children in different parts of the world. Using Arabic digits commonly employed by many cultures, the ability to make simple numerical comparisons were compared for Chinese and English native speakers. Despite the identical input and tasks used, quite different networks of brain areas were used by the two groups. English native speakers used the network of parietal and frontal areas discussed above. However, Chinese natives relied on premotor areas not found active for English speakers (Tang et al., 2006). These fMRI findings indicate the different neural systems may be involved in dealing with very simple numerical tasks. We do not know the reasons for these differences, but if they arise from training, it may provide a way to improve the understanding of quantity, which has shown to be deficient in low-SES preschool children (Griffin et al., 1995).

As the tasks were increased in difficulty by requiring addition as well as comparison the English natives speakers used language areas, as had been reported previously for exact calculation (Dehaene et al., 1999). In addition, English speakers activated limbic areas related to anxiety. However, Chinese native speakers did not show activation of language areas, nor of areas related to anxiety and negative affect. Whether the differences between Chinese speaking and English speaking children lies in genes, early experience, educational method of some other difference is yet to be determined and is of great importance because of the strong advantage which various Asian groups have shown in elementary arithmetic tests. The imaging results show that the brain networks used by Chinese children differ from English speakers suggesting that more than mere effort is involved, but has not yet provided a clear reason for the difference.

## INFLUENCE OF SES

There is wide agreement that learning of arithmetic operations depends on the early skill in the ability of children to understand quantity (Siegler, 2009). Significant differences in the numerical proficiency of preschoolers and kindergartners from different SES backgrounds have been described on a wide range of tasks such as reciting the digits, counting sets of objects, counting up or down from a given number other than 1, recognizing written numerals, adding and subtracting, comparing numerical magnitudes, and the ability to describe thinking and explain ideas in the context of mathematical problem solving (Crane, 1996; Pappas et al., 2003; Ramani and Siegler, 2008).

Studies using a program called Rightstart (Griffin et al., 1995) indicated that children from low-SES homes were at high risk for failure in elementary school arithmetic, but training in numerical quantity before the start of school could greatly reduce this deficit. Manual and computerized exercises based on this concept have been developed for young children. For instance, Ramani and Siegler (2008) have tested the prediction that playing linear number board games should enhance children’s numerical knowledge by applying an intervention in which low-income preschoolers played a game for 1 h. Results showed increased proficiency on several numerical tasks (i.e., magnitude comparison, number line estimation, counting and identification). They have also verified that these gains remained 9 weeks after playing and that home experience playing number board games were associated with numerical knowledge, suggesting that playing these games with children from low-SES or poor homes would increase their numerical knowledge. In addition, Wilson et al. (2009) tested the effectiveness of an adaptive game designed to improve number sense in a sample of low-SES kindergartners. They have found that children improved their numerical competences in comparison of digits and words suggesting a change only in one aspect of the number sense competences (i.e., access).

## SUMMARY

The study of attention, literacy and numeracy all point to common roots of school success in the experiences of infancy (Blair and Razza, 2007). Of course as has always been thought the preschool period is important for preparing the child for a successful school experience. We now have many more studies of how these advances arise in the shaping of brain networks. Explicit or implicit training in attention at preschool level may foster the learning of wide variety of skills acquired in school including literacy and numeracy (Posner and Rothbart, 2007a). Brain-oriented research points to both the specific experiences needed and methods to assay whether they have been achieved, as for example the adaptation of the approaches designed by Ramani and Siegler (2008) and Wilson et al. (2009) for low-SES kindergartners, to the classroom context.

## OTHER CURRENT APPROACHES

Several publications reviewing the role of SES and poverty in physical, cognitive, and socioemotional development have been published (Hackman and Farah, 2009; Lipina and Colombo, 2009; Evans and Kim, 2010; Raizada and Kishiyama, 2010). For example, from a developmental cognitive perspective, Bradley and Corwyn (2002) provide an overview of the association between SES and children’s well-being in cognitive, socioemotional, and health development domains. In their approach, the cognitive domain was considered in terms of school achievement, language proficiency and IQ; and the socioemotional domain was approached in terms of symptoms of psychiatric disturbance (e.g., internalizing/externalizing behaviors, use of substances) and maladaptive social functioning.

From a combined developmental and sociological perspective, Conger and Donnellan (2007) have addressed the relationship between SES and health disparities, over the life span. They have analyzed three general theoretical approaches aimed at providing explanations for SES and development associations: the social causation, social selection, and interactionist theories. Duncan and Magnuson (2012), based on recent evidence involving associations between manipulation of family income and children cognitive functions (Duncan et al., 2011), have noted the need to increase the sophistication in the measurement and modeling of SES.

From a health perspective, Evans and Kim (2010) have approached the analysis of SES gradients in terms of exposure to multiple risk factors (e.g., low housing and neighborhood quality, pollutants, toxins, crowding, and noise) that vary with SES. Walker et al. (2011) have reviewed the inequality between groups in developing countries that originate in early adverse experiences. They describe the impact of risk factors (e.g., low cognitive stimulation, stunting, iodine and iron deficiencies, intrauterine growth restriction, malaria, lead exposure, HIV infection, maternal depression, institutionalization, exposure to social violence) with the aim of providing priorities for intervention programs and policies. Mathews and Gallo (2011) attempt to revise psychological theories of SES and physical health, by reviewing psychobiological (i.e., biomarkers, neurotransmitters) and psychosocial factors (i.e., stress and distress).

Using an interdisciplinary perspective that integrates economics, neuroscience, and developmental psychology, Heckman (2006, 2008) considers the rates of return to human capital investment for low-SES children. In his framework, skill formation follows a hierarchical order in which later attainments build on earlier foundations, the author argues that developmental disadvantage arises more from lack of early family stimulation than from the lack of financial resources, so late remediation strategies are not effective.

All these approaches point to the following factors as mediators between SES/poverty and socioemotional and cognitive development: nutrition, access to health care, housing, stimulating cognitive materials and experiences, parent expectations and styles, teacher attitudes and expectations, allostatic load (see below), and health-relevant behaviors.

Recent reviews have discussed SES related to neurocognitive differences. For example, Hackman and Farah (2009) and Lipina and Colombo (2009) have reviewed studies in which behavioral, electrophysiological, and neuroimaging methods have been used to characterize SES disparities in neurocognitive functions. Language and cognitive control showed the most sensitivity to SES. Hackman et al. (2010) examined pre- and post-natal levels of stress, the role of parental care in the development of hippocampal structure and function. The epigenetics of regulation of the HPA axis and the capacity of home environment to stimulate cognition are candidate mechanisms by which SES influences brain development.

McEwen and Gianaros (2010) focused their review on the links between stress-related processes in the social environment and the brain (mainly in adults). The authors have illustrated the joint roles of amygdala, hippocampus, and prefrontal cortex as the brain systems mediating allostatic processes.

Finally, Raizada and Kishiyama (2010) have focused their review on the open research opportunities in the area, and the importance of integrating the neuroimaging dimension to behavioral approaches in the study of how SES disparities influences cognitive and socioemotional development, and intervention efforts as well.

## FUTURE DIRECTIONS

Biological and psychosocial risk factors associated with low-SES and poverty conditions are related with inequalities in child cognitive and socioemotional development that poses a threat to educational attainment and adult productivity worldwide (Heckman, 2006; Walker et al., 2011; Marmot et al., 2012). Low-SES and poverty can have profound effects on the brain and body, and thus influence both mental and physical health.

Policies and interventions can affect neuroplasticity. Emerging translational animal and human research link poverty to neurobiological pathways through changes in gray and white matter (McEwen and Gianaros, 2010). We are at the very start of developing interventions that may aid in improving this situation. In the fields of attention, literacy and numeracy we have reviewed interventions using classroom and individual computer exercises that have proven useful in some low-SES and poor populations.

Much remains to be done to establish the efficacy and improve these interventions. Neuroscience studies and intervention need to consider the complexity of SES as the social sciences have proposed (Evans and Kim, 2010; Mathews and Gallo, 2011).

Another issue that neuroscience should take in consideration is a comprehensive approach to development in terms of theories from other disciplines. Recently, Rao et al. (2010) have made such an effort by using a longitudinal data set including ecologically valid in-home measures of early experience during childhood, and structural brain imaging during adolescence, and have found that parental nurturance at age 4 predicted the volume of the left hippocampus in adolescence. In addition, the association between hippocampal volume and parental nurturance disappeared at age 8, suggesting the existence of a sensitive developmental period for brain maturation. Finally, as Crone and Ridderinkhof (2011) have recently addressed “little headway has been made toward understanding how brain growth maps onto mental growth during child development.” In their review, these authors have aim at bridging and integrating recent human brain maturation findings with the conceptual thinking of theorist in the behavioral tradition of studying cognitive development. In such a context, developmental research in the area of self-regulation could serve as a reference point for understanding the relation between brain and mental development.

It is our hope that this paper may help to enhance our current knowledge. Research could encourage both parents and those responsible for public education to put more emphasis on preschool and early elementary education and to foster their task of ensuring the educational future of the world’s children.

## Conflict of Interest Statement

The authors declare that the research was conducted in the absence of any commercial or financial relationships that could be construed as a potential conflict of interest.
